# Peer Review of “Patient Recommendations for the Content and Design of Electronic Returns of Genetic Test Results: Interview Study Among Patients Who Accessed Their Genetic Test Results via the Internet”

**DOI:** 10.2196/38486

**Published:** 2022-05-31

**Keywords:** user-centered design, genomic medicine, patient portals, electronic health records, return of results, bioethics, EHR, genetics, genetic testing, patient preferences, design, human factors

*This is a peer-review report submitted for the paper “Patient Recommendations for the Content and Design of Electronic Returns of Genetic Test Results: Interview Study Among Patients Who Accessed Their Genetic Test Results via the Internet.”*

## Round 1 Review

### General Comments

This paper [1] covers the design and content of test results, specifically genetic test results, that are reported to patients via patient portals or direct communications. It is a qualitative study regarding these two components, and it focuses on an area that is rarely covered when discussing how to communicate test results to patients. Based on that, it adds a nice component to the literature that is becoming more recognized as an important factor in patient engagement and patient communication.

### Specific Comments

As it is a small study with a limited population, the generalizability is somewhat limited. However, it opens the door for additional studies as well as the possibility of piloting some of the design recommendations from this study in a more applied milieu and incorporating both usability and functionality statistics, as well as the qualitative component, for a full picture of how best to interact with patients and provide them pertinent information.

#### Major Comments

1. In the final paper, I would recommend not including the quoted comments from the qualitative interviews. I would put those in the supplemental materials, as they are interesting, but they do not add that much to the paper itself.

#### Minor Comments

1. One area that is mentioned but not emphasized is the extension of the results of this qualitative study to the communication of nongenetic tests to patients. The same sort of principles should apply in terms of the cover sheet and the detailed explanation. Some of us already do this with our patients, but an extension of this study would allow some evidence to support that practice.
2. It would be nice to expand the study to include both nongenetic test results and diagnostic imaging results in terms of the design, content, and functionality of the results presentation.
3. An additional study would be looking at optimizing results presentation and content for smartphones versus computers or tablets. There may be a way to optimize the presentation of the data so that patients could more easily see the data on the smartphone form factor. That is an area for future study.
